# Algorithmic identification of atypical diabetes in electronic health record (EHR) systems

**DOI:** 10.1371/journal.pone.0278759

**Published:** 2022-12-12

**Authors:** Sara J. Cromer, Victoria Chen, Christopher Han, William Marshall, Shekina Emongo, Evelyn Greaux, Tim Majarian, Jose C. Florez, Josep Mercader, Miriam S. Udler

**Affiliations:** 1 Diabetes Unit, Endocrine Division, Massachusetts General Hospital, Boston, Massachusetts, United States of America; 2 Department of Medicine, Harvard Medical School, Boston, Massachusetts, United States of America; 3 Programs in Metabolism and Medical and Population Genetics, Broad Institute of MIT and Harvard, Cambridge, Massachusetts, United States of America; 4 Northeastern University, Boston, Massachusetts, United States of America; 5 Center for Genomic Medicine, Massachusetts General Hospital, Boston, Massachusetts, United States of America; University of Botswana School of Medicine, BOTSWANA

## Abstract

**Aims:**

Understanding atypical forms of diabetes (AD) may advance precision medicine, but methods to identify such patients are needed. We propose an electronic health record (EHR)-based algorithmic approach to identify patients who may have AD, specifically those with insulin-sufficient, non-metabolic diabetes, in order to improve feasibility of identifying these patients through detailed chart review.

**Methods:**

Patients with likely T2D were selected using a validated machine-learning (ML) algorithm applied to EHR data. “Typical” T2D cases were removed by excluding individuals with obesity, evidence of dyslipidemia, antibody-positive diabetes, or cystic fibrosis. To filter out likely type 1 diabetes (T1D) cases, we applied six additional “branch algorithms,” relying on various clinical characteristics, which resulted in six overlapping cohorts. Diabetes type was classified by manual chart review as atypical, not atypical, or indeterminate due to missing information.

**Results:**

Of 114,975 biobank participants, the algorithms collectively identified 119 (0.1%) potential AD cases, of which 16 (0.014%) were confirmed after expert review. The branch algorithm that excluded T1D based on outpatient insulin use had the highest percentage yield of AD (13 of 27; 48.2% yield). Together, the 16 AD cases had significantly lower BMI and higher HDL than either unselected T1D or T2D cases identified by ML algorithms (*P*<0.05). Compared to the ML T1D group, the AD group had a significantly higher T2D polygenic score (*P*<0.01) and lower hemoglobin A1c (*P*<0.01).

**Conclusion:**

Our EHR-based algorithms followed by manual chart review identified collectively 16 individuals with AD, representing 0.22% of biobank enrollees with T2D. With a maximum yield of 48% cases after manual chart review, our algorithms have the potential to drastically improve efficiency of AD identification. Recognizing patients with AD may inform on the heterogeneity of T2D and facilitate enrollment in studies like the Rare and Atypical Diabetes Network (RADIANT).

## Introduction

Diabetes mellitus affects approximately 37.3 million individuals in the United States [1]. Although most patients with diabetes are diagnosed with type 1 diabetes (T1D) or type 2 diabetes (T2D) [2], some patients’ diabetes phenotypes do not fit “classic” descriptions of these conditions and may be considered atypical if another potential cause of their diabetes (e.g. medication-induced or monogenic) cannot be determined [3]. Further work is needed to refine diagnosis in patients with atypical diabetes (AD) and improve clinical care.

The National Institutes of Diabetes and Digestive and Kidney Diseases recently launched the Rare and Atypical Diabetes Network (RADIANT), a multi-site clinical study that is enrolling patients across the United States with atypical, as yet uncharacterized forms of diabetes, with the goal of improving clinical and genetic characterization of AD [4]. In the context of RADIANT, AD has been considered diabetes without classic features of known forms of diabetes, including T1D, T2D, medication-induced, pancreatic due to a known precipitant, and maturity onset diabetes of the young (MODY). RADIANT aims to recruit approximately 400 participants with suspected AD every year from fifteen sites nationwide. Electronic health records (EHR) represent a rich source of clinical data which may be used to identify patients with unrecognized AD; however, given the complexity in identifying patients with AD, manual chart review is ultimately required to confirm AD status and RADIANT eligibility. Without significant pruning of the available medical records in order to prioritize patients with likely AD for further review, identifying eligible patients would not be feasible due to the high volume of charts with low overall percentage yield of this rare phenotype. Therefore, we were interested in mining EHR data to efficiently identify patients with possible AD for recruitment in studies such as RADIANT. We hypothesized that methods to narrow the list of potential atypical cases could improve the percentage yield of AD patient identification and reduce the demand for labor-intensive manual chart review.

In this study, we aimed to create a high-throughput EHR-based method for identifying individuals with likely uncharacterized forms of AD. We specifically aimed to identify one phenotype of AD: individuals with non-insulin-dependent diabetes and no history of obesity, metabolic syndrome, or other known cause of insulin resistance, which we have termed “insulin-sufficient, non-metabolic” (ISNM) diabetes. Here, we apply this method to EHR data from a large integrated healthcare system-associated biobank, the Mass General Brigham (MGB) Biobank, and determine percentage yield of AD cases, as well as clinical and polygenic characterization of the confirmed AD cases.

## Methods

### Data source and study population

The study population was individuals enrolled in the MGB Biobank through March 2020; the MGB Biobank includes 114,975 individuals who consented to the use of their clinical and genetic information from EHR for scientific discovery [5]. Approval for this analysis of Biobank data was obtained by the Mass General Brigham Institutional Review Board, Protocol numbers 2016P001018 and 2020P000116.

The Biobank has established multiple “curated phenotypes” for common diseases based on machine learning (ML) and natural language processing (NLP) of structured and unstructured clinical data, including lab values, vital signs, ICD codes, medications, and free text in clinical notes [6, 7]. For example, the ML phenotype algorithm for T2D includes ICD codes for T1D and T2D, hemoglobin A1c (HbA1c) values, medication prescriptions, use of insulin syringes or glucose strips, and NLP mentions of key words related to diabetes or secondary causes of diabetes (e.g., “hormones”) [7]. These ML phenotype algorithms were validated in a subset of patients against the gold standard of chart review by a trained, board-certified nurse. The validated ML algorithm for T1D (78% sensitivity, 90% positive predictive value (PPV), 97% area under the receiver operating curve (AUC)) identified 725 individuals with likely T1D [6]. The validated ML algorithm for T2D (88% sensitivity, 90% PPV, 98% AUC) identified 7,147 individuals with likely T2D [7]. We refer to these individuals as the ML T1D and ML T2D groups, respectively.

### Atypical diabetes algorithm design

**Fig 1:**
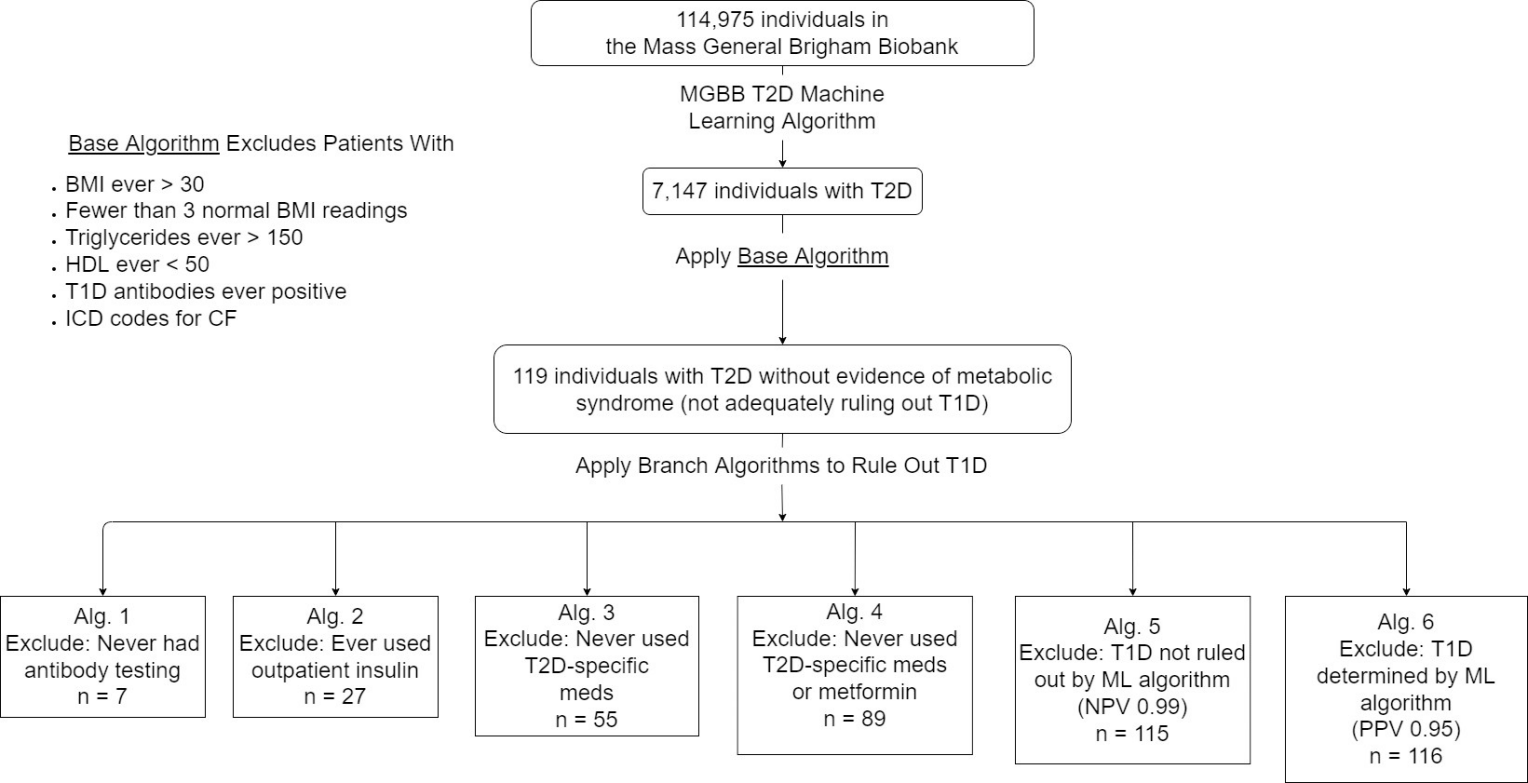
Flow diagram–base and branch algorithms for identifying patients with possible atypical diabetes (AD) in the Massachusetts General Brigham (MGB) Biobank. MGBB: Mass-General Brigham Biobank; T2D: type 2 diabetes; BMI: body mass index; HDL: high-density lipoprotein; T1D: type 1 diabetes; NPV: negative predictive value; PPV: positive predictive value.

Starting with the 7,147 individuals in the ML T2D group, we developed a “base algorithm” to filter out individuals with “typical” T2D who ever had evidence of metabolic syndrome, cystic fibrosis-related diabetes (CFRD), or laboratory-demonstrated autoimmune diabetes (Fig 1). Specifically, the algorithm removed individuals with HDL values ever less than 50 mg/dL, triglyceride values ever greater than 150 mg/dL, or BMI values ever greater than 30 kg/m^2^, without requirement for these criteria to be met concurrently, to rule out those with metabolic syndrome or insulin resistance as a likely contributor to diabetes onset. Individuals with fewer than three normal BMI lab values in the EHR were excluded to ensure reliable engagement with the healthcare system over time and thus accuracy of phenotype data. Finally, individuals were excluded if they had ICD codes for cystic fibrosis or ever had any positive T1D autoantibodies, including glutamic acid decarboxylase 65-kilodalton isoform (GAD65), islet antigen 2 (IA2), and zinc transporter 8 (ZnT8).

While we removed patients with positive autoantibodies in the “base algorithm” to filter out T1D cases who were inappropriately included in the ML T2D starting group, many patients never had antibodies checked or could have tested negative but otherwise fit clinically with typical T1D. We therefore next developed and evaluated six “branch algorithms” as different possible methods to rule out patients with likely T1D (Fig 1), leading to six overlapping cohorts of potentially atypical cases.

- The first algorithm excluded those who did not have documented negative antibody testing such that they could potentially have unreported positive antibodies.
- The second algorithm excluded those who had ever used insulin in the outpatient setting.
- The third algorithm excluded those who had never received any T2D-specific medications (sulfonylureas, meglitinides, thiazolidinedione, dipeptidyl-peptidase 4 inhibitors, alpha-glucosidase inhibitors, glucagon-like peptide-1 receptor agonists [GLP1-RA], and sodium-glucose cotransporter-2 inhibitors [SGLT2i]), as of early 2020; GLP1-RA and SGLT2i received FDA approval for usage by patients without T2D in 2021 and 2020, respectively.
- The fourth algorithm excluded those who had never used either a T2D-specific medication or metformin.
- The fifth algorithm excluded those for whom predicted T1D was not ruled out by a Biobank-validated ML algorithm with negative predictive value (NPV) of 0.99 [6].
- Lastly, the sixth algorithm excluded those with predicted T1D as per a Biobank-validated ML algorithm with PPV of 0.95 [6].

### Atypical diabetes algorithm validation

**Fig 2:**
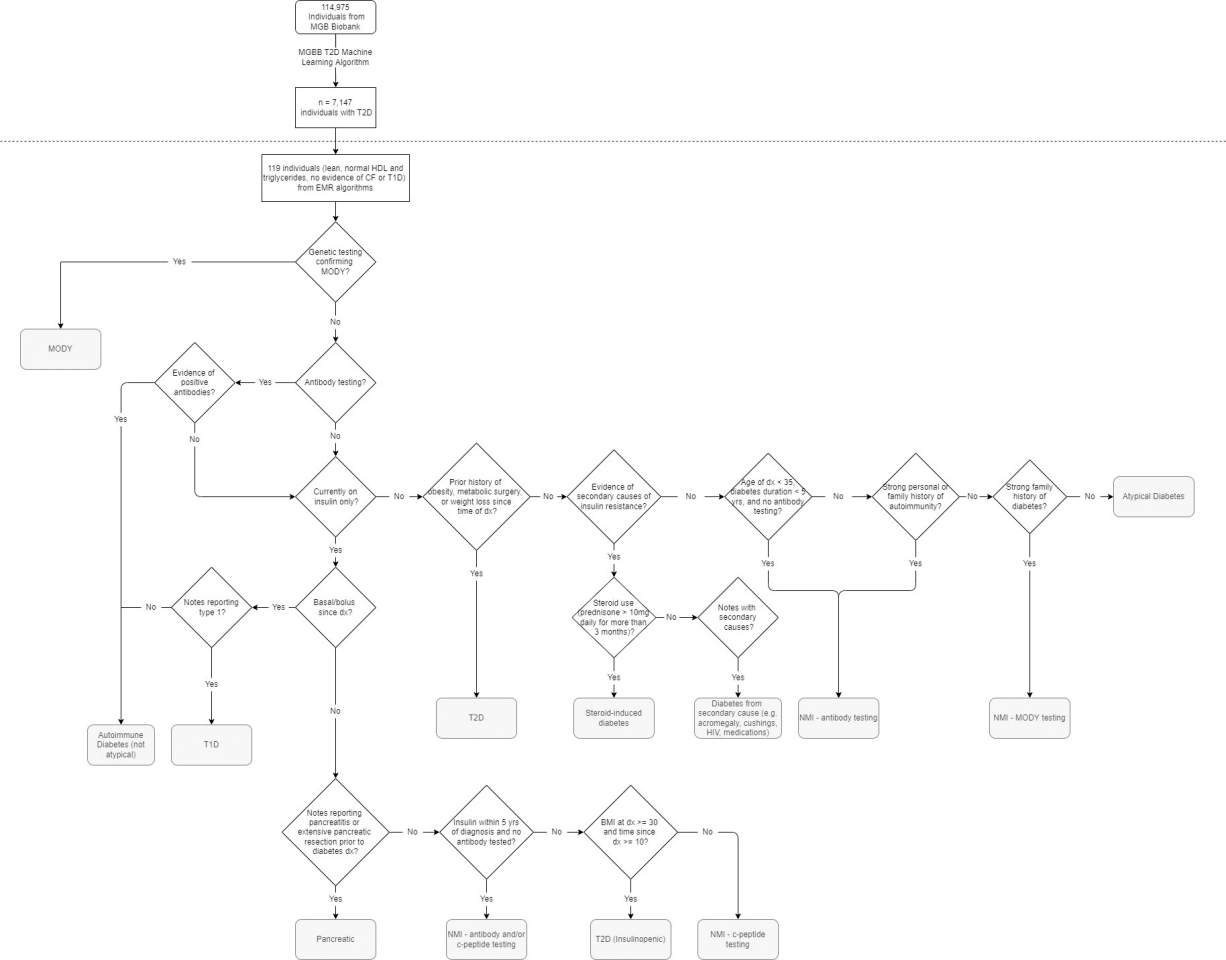
Method for classification of diabetes type. Research assistants summarized and endocrinologists manually reviewed identified charts to classify patients into one of three categories: atypical diabetes (AD), not AD (including typical type 1, type 2, LADA, MODY, pancreatic, steroid-induced, and secondary diabetes), and need more information (NMI). Methodology for classification of diabetes based on clinical data including interpretation of genetic testing, lab values, medication use, and medical history. MGB: Mass-General Brigham; MGBB: Mass-General Brigham Biobank; T2D: type 2 diabetes; HDL: high-density lipoprotein; CF: cystic fibrosis; T1D: type 1 diabetes; EMR: electronic medical records; MODY: maturity-onset diabetes of the young; dx: diagnosis; yrs: years; NMI: need more information; BMI: body mass index.

To test the combined base and branch algorithms, research assistants (authors VC, CH, WM and SE) summarized and two board-certified endocrinologists (authors SJC and MSU) manually reviewed the lab results, anthropometrics, and clinical notes of each patient identified by the algorithm. Classification of diabetes type was based on age of diagnosis, antibody and c-peptide results, genetic testing (MODY), medication use, family history, personal history of metabolic and autoimmune diseases, and disease trajectory (Fig 2). All patients determined by the algorithm to have possible AD were classified into one of the following categories: not atypical (including identifiable known forms of diabetes such as typical T1D; typical T2D; MODY; autoimmune diabetes including latent autoimmune diabetes in adulthood (LADA); pancreatic, steroid-induced, and other secondary forms of diabetes), AD, or indeterminate diabetes type due to incomplete information (Need More Information [NMI]). The NMI category included individuals who were missing needed information to confirm diabetes subclassification, such as antibody and c-peptide testing, family history, or medical history. Given that patients with MODY are a subset of those with ISNM diabetes, patients with enough characteristics that raised suspicion for MODY were classified as NMI, specifically requiring MODY testing. Final determinations of diabetes type were decided by the consensus of the two endocrinologists.

Among those with confirmed AD after manual review, we noticed patterns of phenotypic characteristics and further classified these cases into the following categories: ketosis-prone diabetes (KPD) (specifically antibody negative, β cell positive (A-B+)), mild age-related diabetes (MARD), and ISNM diabetes [8, 9]. Individuals were classified as “KPD,” specifically A-B+, if they had a history of ketosis in adulthood without evidence of autoimmunity or need for long-term insulin use [8]. Individuals were classified as “MARD” if their diabetes onset occurred after a soft cutoff of age 60 and was characterized by a mild, non-progressive course determined by consistent HbA1c values of less than 8% (64 mmol/mol) on 2 or fewer non-insulin diabetes medications over at least five years, suggestive of the diabetes subtype described broadly by Ahlqvist et al. [9].

### Polygenic score assignment

Polygenic risk scores (PRS) were compared between individuals with AD, ML T1D, and ML T2D for whom genetic information was available. In brief, genotyping for approximately 36,000 individuals in the MGB Biobank had been completed at the time of our analysis. Genotyping was performed using three single nucleotide polymorphism (SNP) arrays offered by Illumina (Multi-Ethnic Genotyping Array, the Expanded Multi-Ethnic Genotyping Array, and the Multi-Ethnic Global BeadChip), as has previously been described [10]. Individual SNPs were quality controlled by excluding those with high missing call rate, deviation from Hardy-Weinberg equilibrium, differences in proportions of missingness between cases and controls, or low minor allele frequency. All SNPs were genotyped or imputed with high quality (r^2^ values > 0.95). Individual samples were quality controlled by excluding those with gender discordance, high subject relatedness, high missing call rates, or population structure more than four standard deviations away from the mean of the study population in any of the first four principal components. Phasing was performed with SHAPEIT2 [11] and then imputed with the TOPMed Reference Panel (ref TOPMED) [12] using the TOPMed Imputation Server [13].

Genetic risk of individuals for T1D and T2D was assessing using polygenic risk scores (PRS). A restricted to significant polygenic score (rsPS) for T1D [14] and a global extended polygenic score (gePS) for T2D [15] were generated based on published summary statistics with individual genotypes imputed using the TOPMed reference panel [13, 16, 17] and using PRS-CS [18] and PLINK—score function [19] to generate final scores using allele dosages. All PRS were generated by multiplying a variant’s genotype dosage by its weight. T2D gePS were linearly transformed by setting the minimum value as 0 and shifting the scores into the positive range for ease of interpretation. As both polygenic scores were developed in European populations, and PRS values in non-European populations are systematically skewed, comparisons between groups were restricted to those of European ancestry [20, 21].

### Statistical analysis

Baseline characteristics and outcomes were reported using number and proportion for categorical variables, mean and standard deviation (SD) for normally distributed continuous variables, and median and interquartile range (IQR) for non-normally distributed continuous variables or groups with small sample size. All analyses were performed using R version 3.6.1 [22]. Comparison of clinical measures between groups were performed using the Wilcoxon rank sum test for continuous variables and the Kruskal-Wallis test by ranks for categorical variables with a significance threshold of 0.05.

## Results

Among the 114,975 patients enrolled in the MGB Biobank as of March 2020, mean age was 56.68 years, 64,325 (56.0%) were women, 97,181 (84.5%) were non-Hispanic white, and mean most recent BMI was 28.2 kg/m^2^, and 7,147 (6.2%) were believed to have T2D based on the Biobank machine-learning algorithm (Table 1). After applying a “base algorithm” to remove patients with “typical” T2D with features of metabolic disease, CFRD, and laboratory demonstrated autoimmune diabetes, and the “branch” algorithms to exclude potential T1D cases (see methods, Fig 1), 119 patients (0.1% of Biobank enrollees and 1.7% of individuals in the ML T2D group) with possible AD without features of metabolic syndrome remained. They included 44 women (37.0%) and 96 non-Hispanic whites (80.7%) with mean age 71.0 years, mean most recent BMI 22.7 kg/m^2^, and mean most recent HbA1c 7.1% (54.1 mmol/mol) (Table 1).

**Table 1:** Baseline characteristics by biobank vs. algorithm-identified.

Characteristic	MGB Biobank (n = 114, 975)	Potential AD cases collectively identified by algorithms (n = 119)
Age (years), mean (SD)	56.7 (17.7)	71.0 (11.6)
Gender (Female), n (%)	64,325, 56.0%	44, 37.0%
Race (White), n (%)	97,181, 84.5%	96, 80.7%
Race (Black), n (%)	5,456, 4.8%	7, 5.9%
Race (Hispanic), n (%)	2,830, 2.5%	2, 1.7%
Race (Asian), n (%)	3,056, 2.7%	6, 5.0%
Race (Other), n (%)	2,917, 2.5%	4, 3.4%
Most Recent BMI (kg/m^2), mean (SD)	28.2 (6.4)	22.7 (2.7)
N with T2D (PPV 0.90), %	7,147, 6.22%	
N with T1D (PPV 0.90), %	725, 0.63%	

SD: standard deviation; BMI: body mass index; T2D: type 2 diabetes; PPV: positive predictive value; T1D: type 1 diabetes.

Clinical characteristics and diabetes classification among patients identified collectively by the branch algorithms (comprising six non-mutually exclusive cohorts) are summarized in S1 Table. We took an objective approach to determine the diabetes type of the 119 patients through detailed manual review (Fig 2). Overall, 16 patients (13.4% of all possibly atypical cases) were confirmed by expert review to have AD. The percentage yield of confirmed AD cases of each of the six branch algorithms ranged from 10.9% to 48.2%. The second algorithm (excluding patients who had ever used outpatient insulin) had the highest percentage yield of atypical cases, including 13 of 27 (48.2%) identified patients. Additionally, 15 patients (12.6%) were classified as “need more information,” or requiring further information (e.g., BMI at the time of diabetes diagnosis) or evaluation (e.g., antibody testing) in order to determine diabetes type

Of the 16 patients with AD identified by any of the algorithms, further subclassification was performed based on clinical features (see Methods). One (6.3%) patient was classified as KPD, 7 (43.8%) as MARD, and 8 (50.0%) as ISNM diabetes. The median age of diagnosis was 58 years (46.5–66.0). Clinical characteristics of the patients with AD are presented in Table 2 and S2 Table.

**Table 2:** Demographic and phenotypic characteristics of individuals with atypical diabetes.

Case	Sex	Age	Race	Age at diagnosis	BMI range	HbA1c range	Current treatment regimen	Family History (members)	Phenotypic Pattern
1	M	41	Black	35	23.9–27.8	5.3–11.2	Metformin	Yes (Father, brother, uncle)	A- B+ KPD
2	F	67	White	42	21.0–25.2	6.2–9.1	Metformin, basal insulin, and liraglutide	No	ISNM
3	M	61	White	35	16.8–23.8	7.5–8.6	Metformin, glipizide	Yes (Mother, sister)	ISNM
4	F	62	White	52	23.0–24.0	6.2–6.7	Metformin, glimepiride	No	ISNM
5	M	69	White	58	23.4–27.3	7.0–7.3	Metformin	No	ISNM
6	M	74	White	68	22.1–24	6.1–6.4	Diet	Yes (Father)	MARD
7	F	72	Asian	60	18.5–22.5	5.8–8.8	Metformin, empagliflozin	No	ISNM
8	M	84	Asian	67	19.2–22.2	6.2–7.3	Glipizide	No	MARD
9	M	67	White	62 or earlier	25.6	6.2–6.7	Pioglitazone, metformin (prednisone)	No	MARD
10	M	76	White	70 or earlier	24.1–26.2	5.5–6.2	Sitagliptin	No	MARD
11	F	70	White	65	22.3–25.7	5.5–6.1	Metformin	No	MARD
12	M	89	White	77	17.5–21.8	5.8–6.3	Metformin (discontinued)	No	MARD
13	F	77	White	58	21.8–25.6	5.6–9.4	Diet-controlled	No	ISNM
14	M	68	White	49	22.4–25.9	6.6–8.1	Metformin	Yes (Brother)	ISNM
15	F	83	UNK	67	18.9–21.5	5.3–6.1	Diet	Yes (Brother)	MARD
16	M	54	UNK	44	23.3–26.2	UNK	Metformin	No	ISNM

KPD: ketosis-prone diabetes; ISNM: insulin-sufficient; non-metabolic diabetes; MARD: mild age-related diabetes; UNK = unknown.All individuals identified by the algorithm reported non-Hispanic ethnicity.

We assessed how the patients with AD compared to those in the ML T1D and the ML T2D groups (n = 722 of 725 and 7,028 of 7,147, respectively, excluding individuals identified by the base and branch algorithms; Table 3). Compared to the ML T2D group, the AD group had expected differences related to the algorithm design, with lower median most recent BMI (23.2 vs. 30.7 kg/m^2^, *P* = 6.1×10^−9)^ and higher median most recent HDL (75.0 vs. 46.0, *P* = 1.4×10^−3^). The atypical group also had lower median most recent BMI (23.2 vs. 26.2 kg/m^2^, *P* = 2.9×10^−4^) and higher median most recent HDL (75.0 vs. 58.0, *P* = 4.2×10^−2^) compared to the ML T1D group. Those with AD had statistically lower median most recent HbA1c compared to the ML T1D group (6.5% or 48 mmol/mol vs. 7.6 or 60 mmol/mol, *P* = 6.2×10^−3^) and non-significantly lower HbA1c compared to the ML T2D (6.5% or 48 mmol/mol vs. 7.0 or 53 mmol/mol, *P* = 0.065).

**Table 3:** Comparison of characteristics between atypical, ML T1D, and ML T2D groups.

Demographic and Clinical Characteristics	Atypical (n = 16)	ML T1D (n = 722)	ML T2D (n = 7,028)	atypical vs. ML T1D p-value	atypical vs. ML T2D p-value
Age (years), median (IQR)	69.5 (65.8, 76.3)	**49.3 (35.4, 60.9)**	69.5 (60.6, 76.7)	**1.2E-04**	6.6E-02
Gender (Female), n (%)	6, 37.5%	383, 52.1%	3109, 44.2%	0.22	0.59
Race (White), n (%)	11, 68.8%	609, 84.4%	5328, 75.8%	0.07	0.36
Race (Black), n (%)	1, 6.31	46, 6.4%	777, 11.1%		
Race (Hispanic), n (%)	0, 0.0%	9, 1.3%	290, 4.1%		
Race (Asian), n (%)	2, 12.5%	11, 1.5%	144, 2.1%		
Race (Other or Unknown), n (%)	2, 12.5%	47, 6.5%	489, 7.0%		
Most Recent BMI (kg/m^2), median (IQR)	23.2 (21.7, 23.9)	**26.2 (23.3, 30.0)**	**30.7 (26.9, 35.3)**	**2.9E-04**	**6.1E-09**
Most Recent HbA1c (%), median (IQR)	6.5 (6.2, 7.1)	**7.6 (6.9, 8.8)**	7.0 (6.3, 8.1)	**6.2E-03**	6.5E-02
Most Recent HDL (mg/dL), median (IQR)	75.0 (69.5, 85.8)	**58.0 (47.0, 73.0)**	**46.0 (37.0, 56.0)**	**4.2E-02**	**1.4E-03**
Most Recent Triglycerides (mg/dL), median (IQR)	78.5 (62.5, 99.5)	90.00	130.0 (90.0, 189.2)	0.96	6.7E-02
T2D Polygenic Score	Atypical (n = 6)	ML T1D (n = 196)	ML T2D (n = 2,531)	atypical vs. ML T1D p-value	atypical vs. ML T2D p-value
T2D gePS, median (IQR))*	1.34 (1.30, 1.39)	**1.05** (0.87, 1.23)	1.17 (1.00, 1.34)	**5.5E-03**	5.1E-02
T2D gePS Centile, median (IQR)	87.50 (84.75, 91.00)	**54.50** (30.75, 79.25)	71.00 (47.00, 88.00)	**5.8E-03**	5.3E-02
T1D Polygenic Score	Atypical (n = 6)	ML T1D (n = 191)	ML T2D (n = 2,465)	atypical vs. ML T1D p-value	atypical vs. ML T2D p-value
T1D rsPS, median (IQR)*	12.84 (9.41, 13.78)	13.57 (12.03, 14.95)	10.10 (8.39, 11.82)	0.13	0.19
T1D rsPS Centile, median (IQR)	87.50 (40.00, 94.50)	94.00 (79.50, 99.00)	51.00 (25.00, 77.00)	0.14	0.18

* For the ML T1D and T2D groups, this includes scores only from individuals of European ancestry with genetic information. As the polygenic scores were developed in European populations, they are more reliable in this population, and values in non-European individuals and scores calculated in non-European populations are systematically skewed. All individuals with AD and genetic information available were of European ancestry.

AD: Atypical Diabetes ML: machine learning; T1D: type 1 diabetes; T2D: type 2 diabetes; SD: standard deviation; BMI: body mass index; HbA1c: hemoglobin A1c; HDL: high-density lipoprotein; TGs: triglycerides; gePS: global extended polygenic score; rsPS: restricted to significant polygenic score

**Fig 3:**
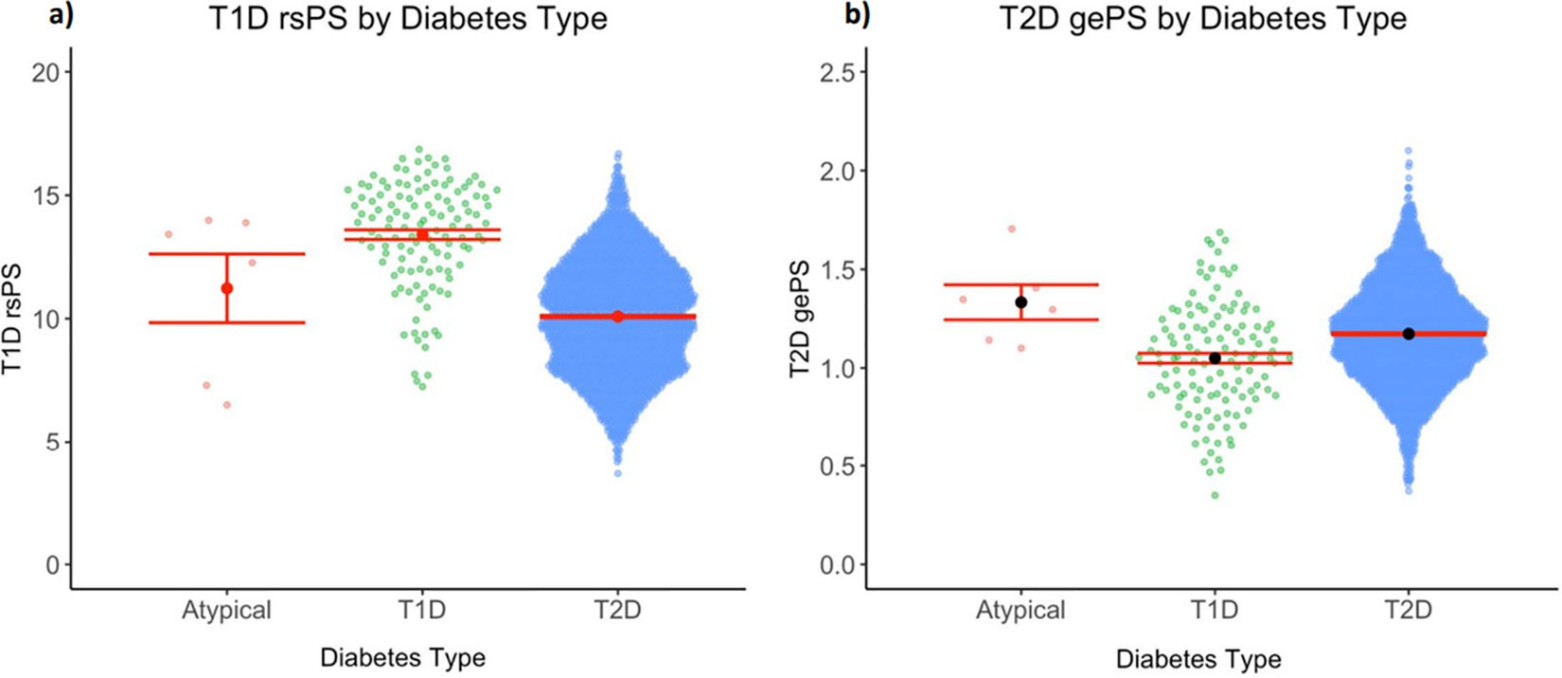
Polygenic scores compared between individuals in the AD, ML T1D, and ML T2D cohorts: (a) T1D rsPS, and (b) T2D gePS. **(a)** Restricted to significant polygenic score (rsPS) for T1D did not differ significantly between the atypical diabetes (AD), machine learning (ML) type 1 diabetes (T1D), and ML type 2 diabetes (T2D) cohorts. **(b)** The global extended polygenic score (gePS) for T2D was significantly higher in the AD group in comparison to the ML T1D cohort.

Genetic information was available for six of the 16 (37.5%) individuals designated as atypical, all of European ancestry. We compared the PRS of the atypical group with those from individuals of European ancestry with genetic information available in the ML phenotype groups (ML T1D n = 196, ML T2D n = 2,531). We confirmed that the median T2D gePS in the ML T2D group was significantly greater than in the ML T1D group (1.17 vs. 1.05, *P* = 3.9×10^−8^), and the median T1D rsPS was significantly greater in the ML T1D vs ML T2D group (13.57 vs. 10.10, *P* < 2.2×10^−16^). The AD group had a significantly greater median T2D gePS than the ML T1D group (1.34 vs. 1.05, *P* = 5.5×10^−3^) and a borderline significantly greater median T2D gePS than the ML T2D group (1.34 vs. 1.17, *P* = 0.051). The median T1D rsPS of the atypical group fell in between those of the ML T1D and T2D groups, without significant differences between groups (Table 3, Fig 3, S1 Fig).

## Discussion

In this study, we developed and tested an algorithm for identifying individuals with AD, specifically an insulin-sufficient non-metabolic diabetes phenotype, in a large EHR database as a potential recruitment strategy for the RADIANT study. A base algorithm identified those with T2D without evidence of metabolic syndrome, CFRD, or laboratory demonstrated autoimmune diabetes. Branch algorithms aimed to further exclude individuals with T1D. Altogether, utilization of EHR algorithms resulted in a 60-fold reduction (from 7,147 with ML T2D to 116 likely atypical candidates) in the number of patients with diabetes fitting our potentially atypical phenotype and requiring manual chart review. The branch algorithm that ruled out T1D by excluding those who had ever used outpatient insulin had the highest percentage yield of AD (48.2%). Patients identified as having AD had distinct clinical and genetic features from an unselected biobank population with either T1D or T2D based on ML algorithms.

The group of 16 individuals with AD identified in our study had significantly higher HDL and lower BMI than the ML T2D group, as expected based on our algorithm designed to identify ISNM diabetes. The atypical group had a numerically lower median most recent HbA1c compared to the ML T2D group and a significantly lower HbA1c than the ML T1D group, suggesting a milder phenotype of diabetes in these individuals, on average. The atypical group also had a significantly higher T2D gePS than the ML T1D groups, and their T1D rsPS fell between those of the individuals with ML T1D and T2D (Table 3, Fig 3, S1 Fig). These genetic results suggest that the individuals with AD identified by these algorithms may have biologic differences as compared to patients with typical forms of diabetes, building upon the idea that forms of diabetes lie on a spectrum, and some individuals with AD may reside somewhere between “typical” T1D and “typical” T2D, or may have features characteristic of multiple forms of diabetes [23]. Regarding the non-significantly higher gePS for T2D among AD cases as compared to those with ML, this finding may be related simply to chance or may signify that individuals who develop diabetes even at lower BMI may have elevated risk for diabetes due to alternate pathways. Notably, clinical genetic testing for MODY was not available for these individuals; however, our AD group was intended to exclude people with MODY, and thus individuals with clinical features consistent with classic MODY (e.g. age of diagnosis before age 35, multigenerational family history) were designated as NMI for MODY testing. Further genetic and phenotypic analysis may help identify causal pathways of atypical forms of diabetes in the future [24].

T2D is a heterogeneous disease, and several studies have proposed models to capture this heterogeneity in broad T2D populations, placing patients on a spectrum of physiologic characteristics or categorizing patients into recognizable subtypes of T2D. For example, the “palette model” presents pathophysiological processes and traits as “base” colors and each individual as a mixture of these colors and molecular pathways that lead to diabetes [25]. Maldonado and colleagues categorized patients with adult-onset diabetes presenting with diabetic ketoacidosis (DKA) into four clinically distinct subtypes of KPD, defined by presence or absence of β-cell autoimmunity and β-cell function [8]. Other studies used clustering methods to identify subtypes of T2D. Ahlqvist and colleagues clustered individuals based on six clinical variables including GAD65 autoantibodies, BMI, and HbA1c to identify five subtypes of diabetes with distinct genetic associations and risks of complications [9]. Our group has previously clustered variant-trait associations for T2D and identified five clusters related to pathophysiological processes of insulin deficiency and insulin action [26]. Studies such as these have made significant strides in understanding the heterogeneity of T2D, and have captured T2D subtypes that may potentially represent previously undescribed forms of AD. Nevertheless, AD remains poorly characterized. Studies focusing on AD, such as the RADIANT study, are needed to improve our understanding of the genotypic and phenotypic landscape of AD. For this reason, methods are needed to identify patients with AD at scale.

The described algorithms allowed for identification of individuals with AD within EHR data including hundreds of thousands of patients. Although this method still requires an element of manual chart review, it can drastically narrow the population under review and speed identification of individuals who may warrant additional clinical testing or qualify for research studies of AD.

This study builds upon earlier efforts which have attempted to identify T1D, T2D, and gestational diabetes from EHR data [27–31]. To the best of our knowledge, the only previous similar studies related to identification of AD in EHR data have not yet been published as full manuscripts but are available in abstract form. One study identified cases of atypical pediatric diabetes in a large pediatric hospital using two separate strategies: 1) designing a questionnaire to be completed by healthcare providers to rule out typical diabetes and 2) developing EHR queries to generate reports of atypical cases (unknown diabetes type, T2D onset before age 10, and antibody negative T1D) [32]. These queries identified 67 individuals (1.0% of 6,676 total diabetes cases) with unknown type of diabetes, 64 individuals (6.8% of 1,142 children with T2D) with T2D onset before age 10, and 38 individuals (5.6% of 680 cases of new-onset T1D) with antibody-negative T1D. Another study developed an algorithm to identify cases of AD that filtered for patients with lean T2D based on ICD codes, anthropometrics, and lab values [33]. They performed manual chart review on 126 out of 208 potentially atypical cases, classifying cases as either atypical or typical T2D. Of the 126 individuals reviewed, 12 out of 111 (10.9%) individuals still living were believed to have AD after expert review.

Our research expands upon prior studies by applying and testing additional filters to rule out T1D, further classifying forms of diabetes into more detailed categories using a more objective classification strategy (Fig 2), and using genetic data for comparison between AD and typical T1D and T2D groups. We additionally report a higher percentage yield of atypical cases from among the individuals undergoing detailed manual chart review (16 of 119 (13.5%) cases overall and 13 of 27 (48.2%) cases using the highest-percentage yield branch algorithm) compared to prior studies, with patterns consistent with previously described forms of AD such as KPD and MARD amongst our group of atypical individuals [8, 9].

The study must be interpreted in light of its observational design. Data was collected by retrospective chart review of information collected and documented during routine clinical care, resulting in a number of patients (n = 15 of 119, 12.6%) with missing information and incomplete diabetes type characterization. Comparisons between groups (e.g., atypical vs. ML T1D populations) do not account for multiple testing; therefore, these analyses should be considered hypothesis-generating. In addition, the atypical group consisted of only 16 individuals, of whom only 6 had genetic information available, and as such, comparisons between the atypical group and ML groups have limited power. This group of atypical patients may represent multiple subtypes of AD and analyzing them monolithically may overlook differences between atypical subgroups; conversely, our algorithms focused on a single phenotype of AD and will not capture all atypical forms of diabetes. Our algorithms may also fail to capture all cases meeting our definition of atypical diabetes, leaving some cases unidentified. Finally, while we have attempted to develop an objective method for classification of AD (Fig 2) and required concordant expert chart reviews, the concept of AD remains poorly defined and is still vulnerable to subjective aspects of interpretation.

In conclusion, we developed a method for the high-throughput identification of individuals with AD, specifically ISNM diabetes without evidence of metabolic syndrome, in EHR data and tested them through detailed, manual chart review. Polygenic risk scores for diabetes differed between identified patients with AD and unselected patients with T1D or T2D. Applying these algorithms across databases may aid in generating cohorts of cases of AD for future studies such as RADIANT.

## Supporting information

S1 Fig Distribution of polygenic scores for type 1 diabetes (T1D) and type 2 diabetes (T2D) among individuals in the AD, ML T1D, and ML T2D cohorts. (TIF)

S1 Table Phenotypic characteristics by branch algorithm. (DOCX)

S2 Table Demographic, phenotypic, and genetic characteristics of individuals with atypical diabetes. (DOCX)
