# Supplementary figures and images for: Algorithmic identification of atypical diabetes in electronic health record (EHR) systems
Source: PLoS One. 2022 Dec 12;17(12):e0278759. doi: 10.1371/journal.pone.0278759 (PMC9744270; doi:10.1371/journal.pone.0278759)

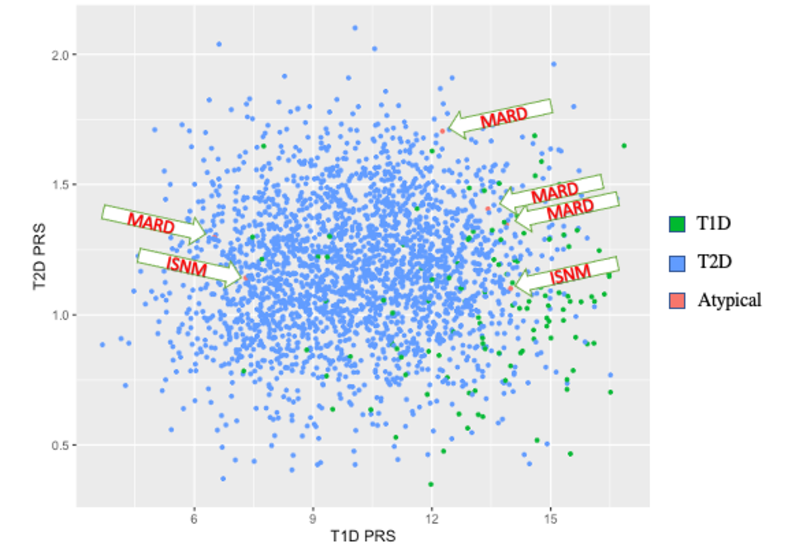

Supplement: S1 Fig — (TIF) [file pone.0278759.s001.tif]
