# Supplementary material for: Algorithmic identification of atypical diabetes in electronic health record (EHR) systems
Source: PLoS One. 2022 Dec 12;17(12):e0278759. doi: 10.1371/journal.pone.0278759 (PMC9744270; doi:10.1371/journal.pone.0278759)
Supplement: S1 Table — (DOCX) [file pone.0278759.s002.docx]

S1 Table: Phenotypic characteristics by branch algorithm.

| Algorithm Name | Alg. 1 (n = 7) | Alg. 2 (n = 27) | Alg. 3 (n = 55) | Alg. 4 (n = 89) | Alg. 5 (n = 115) | Alg. 6 (n = 116) |
| --- | --- | --- | --- | --- | --- | --- |
| Description of Algorithm | Base algorithm* with T1D ruled out by requiring negative antibody testing | Base algorithm* with T1D ruled out by requiring no usage of outpatient ever | Base algorithm* with T1D ruled out by requiring usage of T2D specific oral medications, | Base algorithm* with T1D ruled out by requiring usage of T2D specific oral medications or metformin | Base algorithm* with T1D ruled out by including those without T1D, 0.99 NPV | Base algorithm* with T1D ruled out by excluding those with T1D, 0.95 PPV |
| Atypical by CR, n (%) | 2, 28.6% | 13, 48.2% | 6, 10.9% | 13, 14.6% | 16, 13.9% | 16, 13.8% |
| T1D by Chart Review (CR) , n (%) | 4, 57.1% | 0, 0.0% | 1, 1.8%% | 2, 2.3% | 5, 4.4% | 5, 4.3% |
| T2D by CR, n (%) | 0, 0.0% | 10, 37.0% | 35, 63.6% | 48, 53.9% | 51, 44.4% | 51, 44.0% |
| No Diabetes by CR, n (%) | 0, 0.0% | 1, 3.7% | 1, 1.82% | 1, 1.1% | 8, 7.0% | 8, 6.9% |
| Prediabetes by CR, n (%) | 0, 0.0% | 0, 0.0% | 0, 0.0% | 1, 1.1% | 2, 1.7% | 2, 1.7% |
| Other Types by CR, n (%) | 1, 14.3% | 0, 0.0% | 3, 5.5% | 9, 10.1% | 18, 15.7% | 19, 16.4% |
| NMI by CR, n (%) | 0, 0.0% | 3, 11.1% | 9, 16.4% | 15, 16.9% | 15, 13.0% | 15, 12.9% |
|  |  |  |  |  |  |  |
| Age (years), median (IQR) | 67.0 (53.0, 72.5) | 70.0 (67.0, 81.5) | 74.0 (64.5, 78.5) | 73.0 (65.0, 79.0) | 72.0 (63.5, 79.5) | 72.0 (63.8, 79.3) |
| Gender (Female), n (%) | 4, 57.1% | 11, 40.7% | 19, 34.6% | 32, 36.0% | 41, 35.7% | 42, 36.2% |
| Race (White), n (%) | 4, 57.1% | 18, 66.7% | 45, 81.8% | 70, 78.7% | 93, 80.9% | 93, 80.2% |
| Race (Black), n (%) | 2, 28.6% | 2, 7.4% | 3, 5.5% | 6, 6.7% | 7, 6.1% | 7, 6.0% |
| Race (Hispanic), n (%) | 0, 0.0% | 1, 3.7% | 0, 0.0% | 2, 2.3% | 2, 1.7% | 2, 1.7% |
| Race (Asian), n (%) | 0, 0.0% | 4, 14.8% | 3, 5.5% | 4, 4.5% | 6, 5.2% | 6, 5.2% |
| Race (Other or Unknown), n (%) | 1, 14.3% | 2, 7.4% | 4, 7.3% | 7, 7.9% | 7, 6.1% | 8, 6.9% |
| Most Recent BMI (kg/m^2), median (IQR) | 22.5 (21.5, 24.7) | 23.3 (22.2, 24.1) | 23.2 (21.8, 24.2) | 23.1 (21.3, 24.2) | 23.1 (21.1, 24.2) | 23.1 (21.2, 24.2) |
| Most Recent HDL (mg/dL), median (IQR) | 71.0 (68.0, 80.0) | 74.0 (61.0, 89.0) | 73.0 (60.0, 92.5) | 71.0 (60.0, 89.0) | 71.0 (59.3, 89.0) | 71.0 (59.5, 90.0) |
| Most Recent TGs (mg/dL), median (IQR) | 68.0 (55.0, 96.5) | 68.0 (61.0, 104.0) | 73.0 (59.5, 105.0) | 85.0 (61.0, 104.0) | 71.5 (60.0, 100.0) | 70.0 (60.0, 100.0) |
| Age of Dx (y), median (IQR) | 34.0 (25.5, 48.0) | 61.0 (54.5, 68.5) | 61.0 (50.5, 67.0) | 61.0 (50.0, 68.0) | 61.0 (50.0, 68.0) | 61.0 (50.0, 68.0) |
| Most Recent HbA1c (%), median (IQR) | 6.8 (6.8, 7.5) | 6.5 (6.2, 7.1) | 7.1 (6.5, 7.6) | 7.0 (6.3, 7.5) | 6.8 (6.2, 7.5) | 6.8 (6.2, 7.5) |

*Base algorithm includes individuals with likely T2D by the validated ML algorithm and excludes those with evidence of metabolic syndrome (obesity, dyslipidemia), and cystic fibrosis.
T2D: type 2 diabetes; T1D: type 1 diabetes; ML: machine learning; NPV: negative predictive value; PPV: positive predictive value; SD: standard deviation; NMI: need more information; BMI: body mass index; HDL: high-density lipoprotein; TGs: triglycerides; Dx: diagnosis, HbA1c; hemoglobin A1c.
