# Supplementary material for: Algorithmic identification of atypical diabetes in electronic health record (EHR) systems
Source: PLoS One. 2022 Dec 12;17(12):e0278759. doi: 10.1371/journal.pone.0278759 (PMC9744270; doi:10.1371/journal.pone.0278759)
Supplement: S2 Table — (DOCX) [file pone.0278759.s003.docx]

S2 Table: Demographic, phenotypic, and genetic characteristics of individuals with atypical diabetes.

| Case | Algorithms 1/2/3/4/5/6 | | | | | | Sex | Age | Race | Ethnicity | Age at diag. | BMI range | BMI at diag. | BMI most recent | HbA1c range | HbA1c at diag. | HbA1c most recent | Current treatment regimen | C-peptide (glucose) | Antibodies (GAD65/IA2) | | Years since diag. | Family History (first degree Y/N) | Phenotypic Pattern | T2D gePS | T1D rsPS |
| --- | --- | --- | --- | --- | --- | --- | --- | --- | --- | --- | --- | --- | --- | --- | --- | --- | --- | --- | --- | --- | --- | --- | --- | --- | --- | --- |
| 1 | Y | N | N | Y | Y | Y | M | 41 | Black | Non-His | 35 | 23.9 - 27.8 | 27.8 | 27.8 | 5.3 - 11.2 | 11.2 | 5.3 | Metformin | UNK | 0.00 | 0.00 | 6 | Yes (Father, brother, uncle) | A- B+ KPD | UNK | UNK |
| 2 | Y | N | Y | Y | Y | Y | F | 67 | White | Non-His | 42 | 21.0 - 25.2 | UNK | 23.9 | 6.2 - 9.1 | UNK | 7.8 | Metformin, basal insulin, and liraglutide | 1.00 (UNK) | 0.00 | 0.00 | 25 | No | ISNM | UNK | UNK |
| 3 | N | N | Y | Y | Y | Y | M | 61 | White | Non-His | 35 | 16.8 - 23.8 | UNK | 21.6 | 7.5 - 8.6 | UNK | 7.5 | Metformin, glipizide | UNK | UNK | UNK | 26 | Yes (Mother, sister) | ISNM | UNK | UNK |
| 4 | N | Y | Y | Y | Y | Y | F | 62 | White | Non-His | 52 | 23.0 - 24.0 | 23.2 | 23.0 | 6.2- 6.7 | 6.5 | 6.7 | Metformin, glimepiride | UNK | UNK | UNK | 11 | No | ISNM | UNK | UNK |
| 5 | N | Y | N | Y | Y | Y | M | 69 | White | Non-His | 58 | 23.4 - 27.3 | UNK | 25.1 | 7.0 - 7.3 | UNK | 7.1 | Metformin | UNK | UNK | UNK | 11 | No | ISNM | UNK | UNK |
| 6 | N | Y | N | N | Y | Y | M | 74 | White | Non-His | 68 | 22.1 - 24 | UNK | 23.5 | 6.1 - 6.4 | UNK | 6.2 | Diet | UNK | UNK | UNK | 6 | Yes (Father) | MARD | 1.70 | 12.27 |
| 7 | N | Y | N | Y | Y | Y | F | 72 | Asian | Non-His | 60 | 18.5 - 22.5 | UNK | 21.5 | 5.8 - 8.8 | UNK | 8.8 | Metformin, empagliflozin | UNK | UNK | UNK | 12 | No | ISNM | UNK | UNK |
| 8 | N | Y | Y | Y | Y | Y | M | 84 | Asian | Non-His | 67 | 19.2 - 22.2 | UNK | 19.8 | 6.2 - 7.3 | 7.3 | 6.8 | Glipizide | UNK | UNK | UNK | 17 | No | MARD | UNK | UNK |
| 9 | N | Y | Y | Y | Y | Y | M | 67 | White | Non-His | 62 or earlier | 25.6 | UNK | 23.1 | 6.2 - 6.7 | UNK | 6.3 | Pioglitazone, metformin (prednisone) | UNK | UNK | UNK | 5 | No | MARD | UNK | UNK |
| 10 | N | Y | Y | Y | Y | Y | M | 76 | White | Non-His | 70 or earlier | 24.1 - 26.2 | UNK | 25.2 | 5.5 - 6.2 | UNK | 6.2 | Sitagliptin | UNK | UNK | UNK | 6 | No | MARD | UNK | UNK |
| 11 | N | Y | N | Y | Y | Y | F | 70 | White | Non-His | 65 | 22.3 - 25.7 | 23.9 | 22.3 | 5.5 - 6.1 | 6.00 | 5.5 | Metformin | UNK | UNK | UNK | 5 | No | MARD | 1.30 | 13.89 |
| 12 | N | Y | N | Y | Y | Y | M | 89 | White | Non-His | 77 | 17.5 - 21.8 | UNK | 19.7 | 5.8 - 6.3 | UNK | 6.3 | Metformin (discontinued) | UNK | UNK | UNK | 12 | No | MARD | 1.33 | 13.42 |
| 13 | N | Y | N | N | Y | Y | F | 77 | White | Non-His | 58 | 21.8 - 25.6 | UNK | 24.0 | 5.6 - 9.4 | 9.4 | 6.2 | Diet-controlled | UNK | UNK | UNK | 20 | No | ISNM | 1.10 | 13.99 |
| 14 | N | Y | N | Y | Y | Y | M | 68 | White | Non-His | 49 | 22.4 - 25.9 | UNK | 23.8 | 6.6 - 8.1 | UNK | 7.2 | Metformin | UNK | UNK | UNK | 19 | Yes (Brother) | ISNM | 1.41 | 8.45 |
| 15 | N | Y | N | N | Y | Y | F | 83 | UNK | Non-His | 67 | 18.9 - 21.5 | UNK | 20.7 | 5.3 - 6.1 | UNK | 5.7 | Diet | UNK | UNK | UNK | 16 | Yes (Brother) | MARD | 1.35 | 6.51 |
| 16 | N | Y | N | Y | Y | Y | M | 54 | UNK | Non-His | 44 | 23.3 - 26.2 | UNK | 23.3 | UNK | UNK | UNK | Metformin | UNK | UNK | UNK | 10 | No | ISNM | UNK | UNK |

Diag: diagnosis; Non-His: Non-Hispanic; KPD: ketosis-prone diabetes; ISNM: insulin-sufficient; non-metabolic diabetes; MARD: mild age-related diabetes; Y: yes; N: no
